# Bullying and other risk factors related to adolescent suicidal behaviours in the Philippines: a look into the 2011 GSHS Survey

**DOI:** 10.1186/s12888-022-04085-w

**Published:** 2022-07-04

**Authors:** Hsuan Chiu, Elisabeth Julie Vargo

**Affiliations:** 1 College of Health, Medicine and Life Sciences, Brunel University London,, Uxbridge, UK; 2 Center for Open Science (COS), Charlottesville, Virginia USA

**Keywords:** Adolescents, Bullying, Gender differences, Suicidal behaviour, Philippines, Loneliness

## Abstract

**Backgrounds:**

The present study retrospectively examined gender differences in bullying and suicidal behaviour (ideation, plan, and attempts) as well as associations between selected risk factors and suicidal behaviour among secondary school Filipino students.

**Methods:**

The study used a secondary data set from the GSHS developed by the World Health Organization, which was conducted in the Philippines in 2011. Participants included 5290 Filipino students (male *N* = 2279, female *N* = 2986). A two-tailed Chi-square of independence was used to test for gender differences and a multivariate logistic regression model explored statistical associations between risk factors and outcome variables.

**Results:**

Chi-square results suggested that gender differences were statistically significant for being bullied χ2 (1, *N* = 2384) = 10.6, *p* = .001, experiencing suicidal ideation χ2 (1, *N* = 857) = 61.7, *p* = .000, making suicide plans χ2 (1, *N* = 590) = 10.2, *p* = .001, and suicide attempts χ2 (1, *N* = 674) = 8.4, *p* = .004, with females showing higher vulnerability to examined risk factors. The logistic regression model also suggested that adolescents claiming to have no close friends were three to four times more likely to attempt suicide. Other strong predictors of suicidal behaviours were loneliness and getting in trouble due to alcohol consumption.

**Conclusions:**

Bullying is an independent yet, not the strongest predictor associated with adolescents’ suicidal behaviour in the present study. The strongest predictors of Filipino adolescents’ suicidal behaviours in the 2011 cohort included having no close friends, loneliness, anxiety and getting in trouble due to alcohol use among both genders. Peer and mental health support programmes need to be made available and accessible for adolescents in the Philippines. Considering the increase in suicide rates in 2020/2021 among Filipino young adults due to the Coronavirus pandemic, it is suggested that preventing suicidal vulnerability in adolescence can hinder this occurrence later on in the lifetime.

## Background

Bullying is a prevalent phenomenon among adolescents globally, resulting in physical and mental health concerns and potentially leading to suicide. The impacts of bullying affect individuals in various aspects, including psychologically and behaviourally [1]. The American Psychological Association (APA) [2] defines bullying as a form of deliberate aggressive behaviours that incur harm or inconvenience upon another individual. Bullying generally involves imbalanced power: physical or social control allows a bully to repeatedly victimise a less powerful person and the bullied individual typically has trouble defending him or herself [2]. The aggressive behaviour can be further categorised as physical (e.g., hitting, kicking, or pushing), verbal (name-calling or mocking), social or relational (social exclusion, rumour spreading) [3, 4]. It has been evidenced [5] that bullying evolves around late primary school age and peaks at middle school age.

A recent study [6] conducted in Indonesia using a data set from the 2015 GSHS Survey, found that among 9969 adolescents, 19.9% reported being victims of bullying. Another study conducted in Nigeria [7] found higher bullying rates compared to the US amongst 600 students: 28% reported experiencing bullying while 42% reported bullying others. According to Programmes for International Students Assessment [8], the average percentage of students who reported being bullied globally was 23% among all PISA participating countries. However, the Western Pacific region reported a higher prevalence of bullying, particularly the Philippines (64.9%) and Indonesia (49.1%). Moreover, Sanapo [9] also found that in Western Visayas (a city in the Philippines), approximately 40% of 340 students reported being bullying by their peers.

Different types of bullying are found to be associated with different impacts. A study [10] identified that victims of bullying are prone to internalise problems, suggesting that victims are at a higher risk of experiencing depressive symptoms, anxiety, or loneliness [11, 12]. In contrast, bullies are prone to externalise problems which include being physically aggressive, consume alcohol, abuse substances, and being truant [13, 14]. Cross country studies conducted in China [15], Turkey [16] and Spain [17] demonstrated that both bullying victims and perpetrators had weaker psychological adjustment and greater emotional and behaviour problems.

In addition to this, gender is also found to be associated with different types of bullying. Research [18, 19] showed that boys are more likely to report being a victim of physical bullying, whereas female students reported encountering more psychological or relational bullying, such as name-calling, rumour spreading and being intentionally socially isolated. However, other research found that there is no difference between the types of bullying between genders [20]. This disparity of gender differences in bullying could be explained by cultural, social, and individual differences in how gender and bullying are regarded, which may influence how participants respond [20].

In literature, social support such as parental and peer support are both identified as risk and protective factors. Studies conducted in Europe [10], Australia [21] and China [22] have confirmed the importance of social support, demonstrating that positive parental support and peer support reduce the likelihood of suicidality among adolescents. In contrast, a lack of social support increases loneliness and depressive symptoms [23] resulting in suicidal behaviour. A positive parent–child relationship and parenting style are among the most important known protective factors against adolescent suicidal behaviour. Shaheen et al., [24] identified supportive parenting and family environment as reducing adolescent anxiety levels. By contrast, limits to family cohesion and family connectedness predicated a higher possibility of adolescent mental health issues and suicidal behaviour. Xiao et al., [25] found that among 6063 Chinese adolescents, victims of bullying benefited from perceived social support. This includes positive parental and peer support, which mitigated against internalising distress. Contrary to the positive outcomes of parental support, controlling parents may put children at higher risk of mental distress and suicidal behaviour. For instance, Goschin et al., [26] conducted a systematic review focusing on the impact of parental control and neglect, the study revealed that controlling parents and parental neglect increased mental health distress, hence the potential for suicidal behaviour [26].

The connection between alcohol consumption and suicidal behaviour has also been identified in literature. For example, Sellers et al., [27] found that adolescents who consumed substances such as alcohol or drugs have a higher risk in thinking about or attempting suicide. Peleg-Oren [28] conducted a study in the United States, analysing data of 44, 532 middle school students which revealed a high prevalence of bullying (59%) and that 21% of the students involved in bullying were more likely to use alcohol than those who were not involved (13%). As adolescents are at a critical stage of physical, psychological, and neurobiological development, exposure to alcohol use increases the possibility of drunkenness [29], being physically aggressive, fatal death and suicidality [30].

### The association between risk factors and suicidal behaviour in adolescence

Suicide is defined as a conscious and deliberate self-injurious action with intention to cause one’s death; it can be preceded by ideation (thinking about killing oneself) making a suicide plan and suicide attempt (both non-fatal and fatal) [31]. Suicidal ideation and planning are significant precursors to suicide attempts, suggesting that suicidal ideation precedes a suicide plan, the plan precedes attempts and suicide attempts result in fatal or non-fatal suicide [32]. Although suicide can happen throughout one’s life, adolescence is critical, as nearly 6% of adolescents pass away because of suicide [33]. According to the World Health Organization [34], one-third of incidences of suicide occur among adolescents in low-middle income countries, rendering this phenomenon particularly critical in certain global regions.

Overall, regions with high-income status (e.g., Europe and North America) report a higher prevalence of suicide rates compared to low or low-medium income regions (e.g., Africa and the Western-Pacific region) [34]. According to the global statistics concerning deaths by suicide in 2019 [35], Greenland ranked the highest (53.34 per 100,000 individual), followed by Ukraine (26.34 per 100,000 individual) and Russia (22.77 per 100,000 individual). North African countries such as Egypt, Algeria, Libya (3.55, 3.61 and 4.59 per 100,000 individual) and the Philippines (4.28 per 100,000 individual) ranked the lowest in global statistics [35]. However, one recent study [36] conducted in five South-East Asian Countries (ASEAN), including Indonesia, Laos, Philippines, Thailand, and Timor-Leste found that the Philippines had relatively higher rates of suicidal ideation (11.0%) and suicide plans (10.6%) compared to other participating countries such as Indonesia (5.2%, 5.5%) and Laos (2.9%, 4.3%). Additionally, the Philippines reported an increasing rate of suicide attempts from 12.8% in 2011 to 16.2% in 2015, ranking the highest versus other countries such as Indonesia (3.9%), Laos (1%) and Thailand (13.0%) [36].

One possible explanation is that high-income regions generally have access to standardised youth risk behaviour surveillance such as Health Behaviour in School-aged Children (HSBC) and well-developed education systems that are more acceptable to individuals with mental health concerns and suicidal behaviour. Whereas in low-medium income areas such as the Philippines and Indonesia, suicide is considered a taboo, where the vast majority of the population do not or are less likely to talk about or accept such behaviour [37]. Additionally, both school-based or community-based mental health services may be relatively poorly resourced in low-middle income countries [38, 39] and provide inadequate support and tracking systems, hence, global data may be underreported.

The causes of suicide are complex. One study [40] conducted in a clinical setting identified that suicidal behaviour is a result of the interplay between several biological (e.g., gender and age), social-environmental (e.g., family abuse, sexual abuse, bullying) or psychological (e.g., depression, anxiety, or loneliness) factors. Biological determinants such as gender and age were found to be associated with suicidal behaviour [41–43]. Adolescents of older age groups (ages between 15 -19) are at a higher risk of exhibiting suicidal behaviour [44]. Whilst the fundamental determinants for adolescent suicidal behaviour vary and remain unclear, research has drawn attention to risk behaviour, mental health concerns, social and interpersonal factors as well as a lack of parental support [45].

Gender is believed to be a primary factor affecting mental health and suicide amongst adolescents. In one systematic review [42] with sixty-seven studies, it was revealed that females (aged between 12 – 26) were at a threefold higher risk of experiencing suicide attempts compared to male adolescents, whereas males were at a twofold higher risk than females of dying from suicide. However, Tang et al., [46] conducted a cross-country, population-based study in 83 countries using the Global school-based Health Survey demonstrating that bullying among adolescents is significantly associated with suicidal behaviour (including ideation, creating suicide plans and suicide attempts) across countries, gender and WHO regions. Moreover, Klomeck et al., [47] also conducted a review with a cross-sectional design that suggested a direct correlation between bullying and suicidal behaviour, specifically among bullying victims and bullies, in which both exhibited higher risks of suicidal ideation and suicidal attempts. However, little is known of the association between gender differences, bullying and suicidal behaviour.

In the Philippines, the prevalence of bullying is higher than in other South-East Asian countries [48], as approximately 47.7% of the students reported being bullied. Whilst previous studies examining bullying and suicidal behaviour carried out in the Philippines mainly focused on the prevalence, the correlation between psychosocial risk factors and suicidal behaviour, little research has investigated other sources of risk and protective factors. Moreover, it is relevant to explore suicidal behaviour risk factors in light of the growing suicide rates of the Philippines. The present study employed the Philippines Global School-based Health Survey conducted in 2011 to examine gender differences concerning bullying, suicidal behaviour, as well as the risk factors associated with suicidal behaviour among adolescents. In particular, this study aimed to address the following questions: (1) Does bullying predict suicidal behaviour among adolescents living in the Philippines? (2) Does gender predict bullying and suicidal behaviour and (3) Are there gender differences in risk factors associated with suicidal behaviour among adolescents living in the Philippines?

## Methodology

### Study design and operational variables

This study utilised a quantitative design, involving secondary analysis of existing data from the Philippines Global-School Based Health Survey (GSHS) conducted in 2011. The GSHS is a school-based survey that is widely used across countries including low and low-medium income areas. This survey was developed by the WHO, in collaboration with United Nations Children’s Fund (UNICEF), The United Nations Educational Scientific and Cultural Organization (UNESCO) and the United Nations Joint Programme on HIV/AIDS UNAIDS. The GSHS aims to provide data on adolescents’ physical and mental health, social behaviour and advocates developing resources for school health programmes and policies [49].

The primary independent variables assessed were gender and being bullied. The original question in the survey was QN20 “During the past 12 months, on how many days were you bullied?” Answers ranged from 0 days, 1 or 2 days, 3 to 5 days, 6 to 9 days, 10 to 19 days, 20 to 29 days, and all 30 days. The answers were adapted to dichotomous responses, yes and no, with yes representing any responses other than 0 days. Other variables included being physically attacked (QN15), getting in fights (QN16), loneliness (QN22), anxiety (QN23), no close friends (QN27), having kind and helpful peers (QN32), alcohol consumption (QN35), getting in troubles due to alcohol use (QN39), truancy (QN53), having understanding parents (QN56) and parental control (QN57).

Outcome variables in this study were suicidal ideation (QN24), suicide plans (QN25) and suicide attempts (QN26). Students were asked “During the past 12 months, did you seriously consider attempting suicide?”, “During the past 12 months, did you make a plan about how you would attempt suicide?” and “During the past 12 months, how many times did you actually attempt suicide?” Responses were recorded dichotomously as yes and no.

### Sampling

The 2011 Philippines GSHS survey was conducted at a national level, which comprised a total number of 5290 students. A two-step cluster sampling approach was utilised. In the first step, schools were selected with probability proportional to their enrolment size; next, classes were randomly chosen within the selected schools, and students in the class were eligible to participate in the survey [50].

Definitions of bullying, along with all the other risk factors included in the current study were provided in the introduction of each module of the questionnaire. Bullying was described in the following manner: “*Bullying occurs when a student or groups of students say or do bad and unpleasant things to another student. It is also bullying when a student is teased a lot in an unpleasant way or when a student is left out of things on purpose. It is not bullying when two students of about the same strength or power argue or fight or when teasing is done in a friendly and fun way.*” [51] .The questionnaire was pilot tested on student populations with analogous characteristics of the target population, to ensure the correct understanding of the questionnaire.

The survey was conducted in the schools where students self-administered their responses to each question on a computer scannable answer sheet. Both public schools and private schools were included. According to the Senate of the Philippines [52], the enrolment rates of secondary school in the Philippines in 2011 were at 60% of the entire adolescent population. Despite that the enrolment rate was relatively low in 2011, the school response rate in the study was 97%, the student response rate was 84%, and the general response rate was 82%.

### Data analysis

Data analysis was performed using SPSS software version 26. A two-tailed Chi square test of independence χ2 was carried out to measure whether gender differences and being bullied independently predicted suicidal behaviour. Three multivariate logistic regression models were employed to measure statistical association between selected risk factors including being bullied, physical abuse, mental health, parental and peer support and forms of aggression, with suicidal behaviours (ideation, plan and attempt) as dependent variables. The significance threshold was set at *p*<0.05.

## Results

### Demographic information, prevalence of selected risk factors

Table 1. shows percentages of participants’ demographic information and selected risk factors. Of the total 5290 secondary students, age range varied from 11 years old or younger to 16 years old or older (M=14.5, SD=1.196). The number of students aged between 11 – 13 years old was 1205 (22.8%), and students aged between 14-16 years old were 4044 (76.5%). Female students (*n*= 2986) were 56.4% of the entire sample, male students (*n*= 2279) were 43.1%. Additionally, 47.9% of the students reported being bullied (*n*=2397), 16.6% reported having had suicidal ideation (*n*=863), 11.5% reported having had made a suicide plan (*n*=592) and 12.9% attempted suicide (*n*=678).

**Table 1:** Demographic information and the prevalence of selected risk factors

Measures						
	Female		Male		Full sample	
	n	%	n	%	n	%
Age						
11 – 13 years old	713	23.9	485	21.4	1205	22.8
14 – 16 years old	2253	75.4	1778	78.1	4044	76.5
Being bullied	1420	49.8	964	45.2	2379	47.9
Anxiety	345	11.6	239	10.5	587	11.1
Loneliness	491	16.8	290	13	788	15.2
Being physically attacked	863	29	836	36.9	1709	32.5
Getting in fights	866	29.3	897	39.7	1772	33.8
Alcohol use in the past 30 days	427	14.6	597	27	1031	19.9
Getting in trouble due to alcohol use	164	5.5	213	9.8	380	7.4
No close friends	79	2.7	86	3.8	166	3.2
Having kind and helpful peers	1015	34.2	687	30.6	1706	32.6
Having understanding parents	860	29	555	24.7	1420	27.1
Parental control	1053	35.5	603	26.9	1662	31.7
Truancy	837	28.2	806	35.9	1650	31.5
Suicidal ideation	593	20.1	264	11.6	863	16.6
Suicide plans	374	12.7	216	9.8	592	11.5
Suicide attempts	417	14	257	11.3	678	12.9

### Bullying, suicidal behaviour and gender differences

A two-tailed Chi-square of independence analysis was performed to determine differences in bullying and suicidal behaviour. Results indicated a significant difference of small effect between being bullied and suicidal ideation χ2 (1, *N*=491) = 69.2, *p*=.000, phi=.119, making a suicide plan χ2 (1, ***N***=318) = 27.2, *p*=.000, phi=.075 and attempting suicide χ2 (1, *N*=408) = 85.2, *p*=.000, phi=.131. Gender differences in bullying and suicidal behaviour were also tested. The results identified a significant difference of small effect in gender differences and bullying χ2 (1, *N*= 2384) = 10.6, *p*=.001, phi=.046 gender differences and suicidal ideation χ2 (1, *N*=857) = 61.7, *p*=.000, phi=.109, a suicide plan χ2 (1, *N*=590) = 10.2, *p*=.001, phi=.045 as well as suicide attempts χ2 (1, *N*= 674) = 8.4, *p*=.004, phi=.040. In all analyses, females showed an increased risk compared to their male counterparts.

### Association between identified risk factors and suicidal behaviour

Table 2 and Table 3 respectively represent the logistic regressions of female and male students who reported being bullied, the 11 selected predictors and suicidal behaviours (including ideation, suicide plan and attempts). Both female and male students who reported experiencing bullying were at a higher risk of having suicidal ideation and attempting suicide. Male students who were bullied were at 1.5 times higher risk of thinking about suicide (OR = 1.46; 95% CI [1.07, 1.98], *p*=.016) and 1. 9 times higher risk of attempting suicide (OR = 1.85; 95% CI [1.30, 2.63], *p*=.001) compared to their counterparts reporting not being bullied. Female students who reported being bullied had a 1.5 times higher chance of having suicidal ideation (OR = 1.54; 95% CI [1.24, 1.92], *p*=.000) and 1.6 times higher in attempting suicide (OR = 1.57; 95% CI [1.21, 2.07], *p*=0.001).

**Table 2:** Multivariate logistic regression model predicting suicide behaviour with selected variables. Results from the female cohort

Variables (female)	^a^Suicidal Ideation		^b^Suicide Plans		^c^Suicide Attempts	
	OR (95% CI)	*P* value	OR (95% CI)	*P* value	OR (95% CI)	*P* value
	LL UL		LL UL		LL UL	
Being bullied	1.547 [1.242—1.927]	*p* = .000**	1.299 [0.998—1.689]	*p* = .051	1.571 [1.218—2.027]	*p* = .001*
Loneliness	2.391 [1.866—3.065]	*p* = .000**	1.772 [1.318—2.382]	*p* = .000**	1.895 [1.431—1.511]	*p* = .000**
Anxiety	1.548 [1.156—2.073]	*p* = .003*	1.587 [1.137—2.215]	*p* = .007*	1.245 [0.894—1.736]	*p* = .195
Being physically attacked	0.951 [0.751—1.203]	*p* = .673	1.126 [0.855—1.483]	*p* = .399	0.991 [0.760—1.292]	*p* = .947
Getting in fights	1.270 [1.008—1.600]	*p* = .043*	1.074 [0.812—1.420]	*p* = .617	1.525 [1.77—1.976]	*p* = .001*
Alcohol consumption	2.263 [1.736—2.951]	*p* = .000**	1.892 [1.386—2.582]	*p* = .000**	1.904 [1.411—2.570]	*p* = .000**
In troubles due to alcohol	1.864 [1.269—2.738]	*p* = .002*	2.338 [1.548—3.530]	*p* = .000**	1.870 [1.244—2.811]	*p* = .003*
Having no close friends	1.639 [0.929—2.892]	*p* = .088	4.433 [2.572—7.640]	*p* = .000**	4.145 [2.425—7.084]	*p* = .000**
Having kind and helpful peers	0.839 [0.665—1.058]	*p* = .137	1.032 [0.787—1.354]	*p* = .819	1.025 [0.788—1.332]	*p* = .854
Having understanding parents	0.717 [0.547—0.939]	*p* = .016*	0.730 [0.530—1.006]	*p* = .054	0.661 [0.482—1.906]	*p* = .010*
Parental control	0.734 [0.574—0.939]	*p* = .014*	0.929 [0.695—1.242]	*p* = .619	0.853 [0.644—1.129]	*p* = .267
Truancy	1.362 [1.088—1.705]	*p* = .007*	1.357 [1.039—1.773]	*p* = .025	1.398 [1.804—1.803]	*p* = .010*

*Abbreviations: CI* Confidence Interval, *LL* Lower Limit, *UL* Upper Limit

^a^ Suicidal ideation: χ2 = 260.461, df = 12, *p* = .000

^b^ Suicide plan: χ2 = 151.750, df = 12, *p* = .000

^c^ Suicide attempts: χ2 = 185.852, df = 12, *p* = .000

*Note.* *p < 0.05, **p < 0.001

**Table 3:** Multivariate logistic regression model predicting suicide behaviour with selected variables. Results from the male cohort

Variables (male)	^a^Suicidal Ideation		^b^Suicide Plans		^c^Suicide Attempts	
	OR (95% CI)	*P* value	OR (95% CI)	*P* value	OR (95% CI)	*P* value
	LL UL		LL UL		LL UL	
Being bullied	1.461 [1.074—1.988]	*p* = .016*	1.292 [0.902—1.851]	*p* = .163	1.856 [1.305—2.637]	*p* = .001**
Loneliness	2.497 [1.704—3.659]	*p* = .000**	1.923 [1.224—3.020]	*p* = .005*	1.694 [1.109—2.587]	*p* = .015*
Anxiety	1.307 [0.823—2.075]	*p* = .257	1.528 [0.913—2.555]	*p* = .106	2.631 [1.693—4.088]	*p* = .000**
Being physically attacked	1.465 [1.069—2.008]	*p* = .017*	1.004 [0.690—1.461]	*p* = .982	1.660 [1.167—2.362]	*p* = .005*
Getting in fights	1.229 [0.893—1.690]	*p* = .205	1.697 [1.172—2.457]	*p* = .005*	1.364 [0.956—1.946]	*p* = .087
Alcohol consumption	1.389 [0.989—1.952]	*p* = .058	1.441 [0.969—2.142]	*p* = .071	1.112 [0.753—1.641]	*p* = .594
In troubles due to alcohol	0.995 [0.585—1.559]	*p* = .853	1.471 [1.867—2.469]	*p* = .152	2.572 [1.634—4.049]	*p* = .000**
Having no close friends	1.825 [0.959—3.472]	*p* = .067	3.010 [1.533—5.910]	*p* = .001*	2.717 [1.415—5.217]	*p* = .003*
Having kind and helpful peers	0.724 [0.507—1.033]	*p* = .075	0.671 [0.440—1.024]	*p* = .064	0.828 [0.550—1.224]	*p* = .345
Having understanding parents	0.812 [0.545—1.209]	*p* = .304	0.872 [0.553—1.375]	*p* = .555	1.123 [0.741—1.703]	*p* = .583
Parental control	0.668 [0.450—0.992]	*p* = .045*	0.722 [0.450—1.142]	*p* = .164	0.753 [0.491—1.154]	*p* = .193
Truancy	1.092 [0.797—1.496]	*p* = .586	1.052 [0.728—1.519]	*p* = .789	1.329 [0.939—1.882]	*p* = .109

*Abbreviations: CI* Confidence Interval, *LL* Lower Limit, *UL* Upper Limit

^a^ Suicidal ideation: χ2 = 85.407, df = 12, *p* = .000

^b^ Suicide plan: χ2 = 68.678, df = 12, *p* = .000

^c^ Suicide attempts: χ2 = 138.864, df = 12, *p* = .000

*Note.* *p < 0.05, **p < 0.001

### Suicidal ideation

The overall model of pupils of both genders and suicide ideation was statistically significant compared to the null model, female students (χ2 (12) =260.461, *p*=.000), explained the variance of 15.3% and male students (χ2 (12) = 85.407, *p*=.000) explained 0.9% of pupils thinking about suicide, and correctly predicted overall 81.1% of cases for female students and 88.8% of male students, respectively. Among the 11 identified predictors, the strongest predictor associated with suicidal ideation among male students included loneliness and being physically attacked. Male students who felt lonely were 2.5 times higher likely to experience suicidal thoughts (OR= 2.49; 95% CI [1.70, 3.65], *p*=.000) and those reported being physically attacked were 1.4 times more vulnerable to suicidal ideation (OR=1.46; 95% CI [1.06, 2.00], *p*=.017). Female students who felt lonely and consumed alcohol were significantly associated with suicidal ideation. Female students who felt lonely were 2.4 times more vulnerable to suicidal ideation (OR=2.39; 95% CI [1.86, 3.06], *p*=.000), and those who reported consuming alcohol were at 2.3 times higher risk of thinking about suicide (OR=2.26; 95% CI [1.73, 2.95], *p*=.000). As evidenced in Table 2, selected variables impacted female suicidal ideation overall more significantly than male suicidal ideation.

### Suicide plan

The overall model of female and male students was statistically significant compared to the null model, female students (χ2 (12) = 151.750, *p*=.000) and male students (χ2 (12) = 68.678, *p*=.000), explained that 11% and 8.7% of pupils making a suicide plan and correctly predicted overall percentage of 88% of cases for female students and 92% of male students, respectively. The strongest predictor associated with making a suicide plan comprised a common predictor among both genders: having no close friends. Male students who reported having no close friends were 3 times higher in making a suicide plan (OR=3.01; 95% CI [1.53, 5.91], *p*=.001), female students were 4.4 times higher (OR=4.43; 95% CI [1.38, 2.58], *p*=.000). Male students who felt lonely were 1.9 times higher in planning suicide (OR=1.92; 95% CI [1.22, 3.02], *p*=.005). Female students who reported getting in troubles due to alcohol consumption were 2.3 times likelier to plan suicide (OR=2.33; 95% CI [1.54, 3.53], *p*=.000). Alcohol consumption and loneliness were also strong predictors of female suicide planning.

### Suicide attempts

The overall model of pupils of both genders attempted suicide was statistically significant compared to the null model, female students (χ2 (12) = 185.852, *p*=.000), explained the variance of 12.6% and male students (χ2 (12) = 138.864, *p*=.000) with 15.8% of pupils attempting suicide and correctly predicted overall percentage of 86.8% of cases for female students and 91.2% of male students, respectively. The strongest predictors of male adolescents’ suicide attempts included having no close friends, and anxiety; predictors associated with suicide attempts among female students were having no close friends and alcohol consumption. Male students who reported having no close friends were 2.7 times likelier to attempt suicide (OR=2.71; 95% CI [1.41, 5.21], *p*=.003), female students with no close friends had 4 times higher (OR=4.1; 95% CI [2.42, 7.08], *p*=.000) probability to attempt suicide. Male students who reported having anxiety had a 2.6 times higher risk of suicide attempt (OR=2.63; 95% CI [1.69, 4.08], *p*=.000) and female students who consumed alcohol had 1.9 times higher possibility of attempting suicide (OR=1.90; 95% CI [1.41, 2.57], *p*=.000).

## Discussion

The primary purpose of this study was to examine the association between gender differences in bullying and suicidal behaviour, as well as examine the association between gender differences in the selected risk factors associated with suicidal behaviour among secondary school adolescents in the Philippines in 2011. The overall prevalence of adolescents being bullied was 47.9%; female students who reported being bullied were 49.8%, male students who were exposed to bullying were 45.2%. Overall prevalence of adolescents who responded to having suicidal ideation was 16.6%, making a suicide plan 11.5% and attempted suicide at least once was 12.8%.

The study suggests that Filipino adolescents were vulnerable to thinking about and attempting suicide, and gender played a role in such behaviour in 2011. Male students exhibited more vulnerability to suicide attempts and less vulnerability to thinking about or planning suicide, as expected. Previous literature [53] provides a possible explanation whereas males tend to behave more impulsively in terms of attempting suicide.

As predicted, bullying is an independent predictor linked to adolescents’ suicidal ideation and suicide attempts in both genders. Partially consistent with previous literature, bullying was a significant predictor of suicidal behaviour in the Filipino cohort yet was not as strong as loneliness and the lack of social network. Risk variables related to loneliness and lack of a social network appeared to be stronger indicators of suicidal behaviours. A possible explanation is that individuals who were victims of bullying were progressively ostracised, leading to lower social competencies and lower self-esteem. Hence, bullying victims are more likely to be continuously bullied and socially excluded from friendship, class, or school community, with no or limited support from peers [54].

Potentially, bullying victims who are constantly bullied and isolated are more likely to undergo loneliness, mental health issues and ultimately result in suicidal behaviours. Peers who witness individuals exposed to bullying are less likely to stand out and intervene, despite understanding that bullying is wrong, as acceptance and security within the peer community are very important for adolescents [55]. Moreover, those who stand out for victims of bullying might also become targets themselves, increasing the possibility of being isolated from their original friend groups or the school community [56]. This may result in the same or similar situation as the bullying victims, who will also be disliked or rejected by their peers.

In support to this interpretation, one strong risk factor connected to adolescents’ suicidal behaviour was having no close friends. In the present study, male students were 2.7 times more likely and female students were 4.1 times more likely to attempt suicide when they claimed to have no close friends. This finding is partially consistent with the study conducted by Bearman and Moody [57] who found that among 13456 American adolescents, female students who were intentionally excluded from the class, friendship, or having friendship issues were more vulnerable to suicide, whereas male students were less likely to be affected by their social surroundings. Nevertheless, having no close friends may lead to more distressed feelings of loneliness and isolation [57].

The occurrence of loneliness and anxiety respectively, significantly predicted adolescents’ suicidal behaviour. This finding is in support with previous studies [58–60]. Pupils who reported feeling lonely and anxious were two to three times more likely to experience suicidal behaviours. The transition from childhood to adolescence usually is accompanied by physical, social, and psychological changes. The challenges that individuals encounter also contribute to the possibility of experiencing psychological distress ultimately resulting in possible suicidal behaviour [33, 61, 62].

Alcohol consumption in the present study was found to associate with suicidal behaviour of female students. This finding is partially in line with Page et al., [63] who analysed data drawn from the 2008 GSHS in four countries (the Philippines, China, Chile, and Namibia) comprising 30,851 adolescents. Results from this study suggested that both female and male adolescents who had experienced psychosocial distress such as loneliness, anxiety, worry and make a suicide plan were more likely to engage in substance use such as alcohol. Individuals tend to use substances such as alcohol as a coping mechanism to alleviate negative feelings and psychological distress [55]. In another study [64] it has been evidenced that female adolescents in South-East Asian countries, including the Philippines, who consumed alcohol may come from poor family backgrounds, have poor life satisfaction and use other drugs such as tobacco and illicit drugs. This is in support with literature [65–67], which describes the relationship between alcohol consumption, quality of life and mental health conditions as intertwined and often compounding. Unlike Page et al., [63], male students in the present study were not likely to engage in suicidal plans and attempts due to alcohol use.

Similar to alcohol consumption, getting in trouble due to alcohol use was significantly associated with suicidal behaviour of females but only suicide attempts of males. Alcohol misuse usually is associated with immediate and long-lasting threats to adolescents’ development, including dependency and addiction [68], as well as co-occurrence of aggressive behaviour (e.g., fighting) [60], intentional and unintentional injuries [61], homicides and suicides [60]. Consuming alcohol may reduce inhibition, increase impulsivity [69] and risk-taking behaviours [70]. With little education provided to adolescents regarding the impacts of alcohol use on physical and mental health and limited restrictions on adolescents’ alcohol consumption implemented in the country [71, 72], misuse and related misconduct are likelier and can lead to increased mental health concerns and suicidal behaviour.

Truancy is also found to be associated with suicidal ideation and suicide attempts among female students in the present cohort. The reasons for adolescents being truant varies from the individual to the national level. A report conducted in 2008, 2014 and 2017 in the Philippines evidenced the primary reasons for secondary school disengagement in school life. These were lack of personal interest, high cost of education, employment and other reasons (e.g., marriage, housekeeping or school records) [73]. The report further illustrated gender differences in truancy: male students generally left school because of the lack of personal interest in schooling, whereas female students left school due to the high cost of education, for employment and marital commitments. Whilst reasons vary, being away from school is not only an indicator of weakened social bonds, but can also lead to adolescents living in unstructured and unprotected environments, exposing them to risky and harmful behaviours [74–76].

Parental control was identified as a risk factor for suicidal ideation among both genders. Moreover, having understanding parents was associated with suicidal ideation and suicide attempts among female students, evidencing its lack of efficacy in protecting females from these behaviours. The quality of the parent-relationship has been identified as both a possible protective and risk factor in adolescents in both clinical and community samples. It has been evidenced that family cohesion and connectedness can have a positive impact on the parent-child relationships across countries [77] and reduce suicidal behaviour [78]. On the other, large sampling designs [79, 80] however, have demonstrated that parental control can escalate adolescent suicidal behaviour, particularly among girls.

In the present study, the age cohort of participants and parenting styles may have determined outcomes. Adolescence is a critical stage where individuals are seeking to be more independent, while needing the guidance and support from parents. Parenting styles may be influenced by culture, religion, and the community, thus parenting practices and parent-child relationships are also affected [81]. For instance, in the context of the Philippines, the impact of parenting styles often differs between sons and daughters [82]. Hock et al., [83] suggested that in the Philippines, parents tend to be stricter with daughters than sons, particularly concerning romantic relationships and sex [84], whereas the strictness of parents towards sons tended to focus on their educational and occupational achievements. A different parenting focus on sons and daughters may influence restrictions of freedom for girls and expectations for boys, and this may lead to the negative impact of parental involvement in suicidal behaviour outcomes [26].

Suggestions and interventions to prevent adolescents’ bullying and suicidal behaviour should involve schoolteachers, parents, and the students themselves. Adults who work with vulnerable adolescents need to ensure that intervention or support is person centred. Twemlow & Sacco [85] suggested that youths need to feel safe, attached and valued in order to learn. Therefore, creating a person-centred environment for youths who are vulnerable would be a crucial essential step to build up a relationship between adolescent, school, and home for interventions. Secondly, adults need to give clear and consistent instructions to the students both at home and in school settings concerning bullying behaviour and the consequences of bullying. Whilst it is possible that one of the parties may not be able to offer such support to students, solving the problem requires both school and home to put in effort and collaborate [85]. Considering results of the present analysis highlighting the pivotal influence of loneliness and the lack of friends in determining suicidal behaviours among Filipino adolescents, it is recommended to envision intervention strategies that promote peer support and supportive connections among adolescents. Programs that focus on promoting peer support can not only increase mutual respect among peers but can also function as an early recognition tool of suicidal behaviours [86, 87].

The present study utilised a 2011 data set drawn from the WHO. Recent data has shown an increasing rate of suicidality among adults in the Philippines [88], especially since the COVID-19 pandemic outbroke and continued in the country [89]. Young adult suicide rates have seen a significant increase particularly among young women [90]. It is possible that, untreated or not addressed suicidal behaviours, particularly thoughts of suicide, or a suicide plan will lead to more vulnerable adults, resulting in the present increase in fatal suicides among young adults in the country. Preventing suicidal behaviours in adolescence can hinder suicide in young adulthood [91]. For adolescents who have witnessed or experienced bullying or suicidal behaviour, it would be imperative to seek immediate support. Individuals who offer support may be schoolteachers, counsellors, parents, friends, as well as healthcare providers such as a psychiatrist, or practitioner psychologist. Despite some psychological factors might be treatable with an early identification of screening or through school or community-based mental health interventions [92], both school and community-based mental health services are insufficient in the Philippines in terms of the numbers of qualified practitioners and mental health services and facilities being distributed unevenly across the country [93].

The strengths of the study include the use of a large representative sample, extensive measures, and rigorous analysis. However, limitations should be considered in the interpretation of results. First, the present study is cross-sectional in nature, therefore it is not possible to determine the causality between suicidal behaviour and other groups of risk factors. Second, self-report questionnaires may result in under-reporting of undesirable behaviour (i.e., alcohol use and suicidal behaviour). The survey used in this study for example, employs questions that cover only the previous 12 months. For those students who experienced relatively lower levels of bullying or suicidal thoughts, it may be difficult to recall the incidences or the timeline of bullying and suicidal thoughts. Acknowledging suicidal behaviour, however, may be more difficult for adolescents in other forms of data collection (e.g., interviews) in countries where suicide is a taboo.

Despite the limitations of the study, the direction of future research could aim to include vulnerable and/or minority groups such as pupils with physical, psychological, or learning difficulties, or members of the LGBT community. These groups are more sensitive and more likely to experience bullying [94] which potentially leads to suicidal behaviours. According to Human Rights Watch (2017), school-attending adolescents who identified as homosexual or bisexual in the Philippines were often marginalised and were often the targets of derision, humiliation, and bullying within the school setting. A qualitative study [95] which explored LGBT students and the problems they encountered in schools evidenced that LGBT students were not only bullied by peers but were also discriminated against by their family or superiors. Another study [96] found that among 185 Filipino, nearly 25% of the participants reported having suicidal ideation and attempted suicide due to the stigma of sex in the country. Additionally, a higher proportion of lesbians and bisexual women experienced suicidal ideation (27.0%) compared to heterosexual females (18.2%); as well as suicide attempts (6.6% versus 3.9%) [97] due to the stressors of being judged for their sexual orientation. Therefore, including such participants in the study could generate informed results that may be of interest to educators, education stakeholders, parents and health practitioners or specialists.

## Conclusion

The study involved a secondary analysis of the Philippines GSHS survey in 2011 which showed that gender differences, bullying and other risk factors were associated with adolescent suicidal behaviour (including ideation, planning and attempts). Female students were at higher risk of engaging in suicidal behaviours, particularly if exposed to risk factors such as bullying, having no close friends, loneliness, and alcohol consumption. Male students were more likely to engage in suicide attempts. The study identified several risk factors in relation to suicidal behaviour. Risk determinants include having no close friends, loneliness, anxiety, getting in troubles due to alcohol use, alcohol consumption, getting in fights, being bullied, being physically attacked and truancy. Generally, the results suggest that bullying does predict suicidal behaviour in Filipino adolescents, but variables related to isolation have a stronger role in predicting these conducts. Consequently, programmes enhancing peer support and healthy friendship networks could be potentially beneficial for suicide prevention. Mental health programmes as well should be made available and accessible in schools and communities in the Philippines.

## Data Availability

The datasets are publicly available in the WHO Non-Communicative Disease (NCD) Microdata Repository, which can be accessed at: https://extranet.who.int/ncdsmicrodata/index.php/catalog/89/get-microdata
